# Beyond pigmentocracy: how country-level ethnoracial configurations shape the effects of skin color on educational inequality in Latin America

**DOI:** 10.3389/fsoc.2026.1786805

**Published:** 2026-05-19

**Authors:** Javier Castillo, Mauricio Salgado

**Affiliations:** 1 School of Government, Universidad Adolfo Ibáñez, Santiago, Chile; 2 Department of Sociology, Universidad de Chile, Santiago, Chile; 3 Faculty of Social Sciences, Universidad Andrés Bello, Santiago, Chile

**Keywords:** inequality, Latin America, race and ethnicity, racism, skin color, social stratification

## Abstract

**Introduction:**

Skin color is a durable status marker in Latin America, yet most comparative evidence remains correlational and offers limited leverage for adjudicating whether observed gradients reflect causal stratification processes, cross-national composition, or measurement error in interviewer ratings.

**Methods:**

Using nationally representative AmericasBarometer 2016–2017 surveys from 18 Latin American countries (*N* = 23,163), we estimate the causal effect of interviewer-rated skin color (PERLA palette; standardized within country) on years of schooling within a counterfactual framework, implementing a partially linear Double/Debiased Machine Learning design that orthogonalizes treatment–outcome estimation from high-dimensional confounding via cross-fitting. We adjust for social origin (maternal education), ethnoracial self-identification, settlement size, age, sex, and country fixed effects, and we explicitly model interviewer-specific rating heterogeneity through interviewer fixed effects to mitigate rater-induced attenuation. We then test contextual moderation using a five-category typology of country-level ethnoracial configurations derived from clustering indicators of phenotypic dispersion and ethnoracial composition.

**Results:**

Under standard identification assumptions (conditional ignorability given controls, overlap, and stable measurement within interviewers), a one–standard-deviation increase in darker skin causally reduces schooling by roughly 0.3–0.5 years; controlling for interviewer fixed effects increases the estimated penalty, consistent with non-classical measurement error biasing naive estimates toward zero. The causal penalty varies systematically across ethnoracial configurations, being strongest in predominantly White/Criollo countries and weakest in Afromestizo ones, with formal tests rejecting homogeneous effects.

**Discussion:**

The study contributes a causal, cross-nationally comparable estimate of colorism in education, demonstrates that interviewer heterogeneity is substantively consequential for identification and magnitude, and shows that historically sedimented national ethnoracial configurations condition the causal impact of skin color on educational attainment in Latin America.

## 1 Introduction

Across world regions, skin color functions as a durable status marker that is systematically associated with stratification processes, even though the historical roots of “light-skin advantage” vary by context and can combine colonial racism, class-coded aesthetics, and contemporary commodification (Dixon and Telles, 2017). In Latin America, where ethnoracial boundaries are often construed as a continuum and “color” is frequently used as an idiom for racialization, ideologies of whitening and mestizaje have historically framed mixture as national identity while simultaneously legitimating hierarchies that place whiteness or proximity to it at the top (Dixon, 2019; Telles et al., 2025; Wade, 1997; Loveman, 2014). Empirically, a convergent finding across Latin American studies using harmonized survey measures, most notably interviewer-rated skin color via the PERLA palette and comparable ethnoracial items, is that darker-skinned individuals (and, in parallel, many Black and Indigenous populations) experience systematic disadvantages in schooling, income, and occupational attainment, including net of social origin controls, although the magnitude of skin color gradients varies across countries and the ordering of ethnoracial categories can be inconsistent (Telles et al., 2015; Bailey et al., 2016; Telles et al., 2025). Recent cross-national work further shows that broad census-style ethnoracial categories can conceal substantial within-category phenotypic heterogeneity, such that skin color captures consequential gradients in education and economic outcomes and helps explain within-group inequality that self-identification alone may miss (Woo-Mora, 2025; Gómez-Echeverry, 2024). In summary, the literature depicts Latin American inequality as neither “color-blind” nor reducible to class alone: rather, skin color and class are jointly implicated in the production and interpretation of disadvantage, including through intergenerational mechanisms and through the difficulty ordinary actors may face in disentangling whether unfavorable treatment is “about” class, skin color, or both (Dixon, 2019; Bailey et al., 2016).

This joint implication of skin color and class has been theorized as operating through multiple, reinforcing mechanisms rather than a single pathway. One mechanism is intergenerational transmission: because skin color is correlated with historically accumulated advantage and disadvantage, part of the observed gradient reflects the inheritance of parental resources, particularly parental education and class position, which mediate access to schooling and subsequent life chances, with the strength of this mediation varying across national contexts (Bailey et al., 2016; Woo-Mora, 2025). A second mechanism is differential treatment under contemporary institutions: even when standard measures of social origins and human capital are held constant, several studies still document residual penalties associated with darker skin, which is consistent with, though not by itself conclusive evidence of, discriminatory processes operating in schools and labour markets, whether through preference-based discrimination or through decision-making under imperfect information (Monk, 2016; Gómez-Echeverry, 2024; Becker, 1957; Phelps, 1972). A third mechanism concerns national racial-formation and classificatory regimes: projects of whitening, mestizaje, and more recent multicultural reforms shape how phenotype is socially read, how ethnoracial categories are institutionalized, and which forms of inequality are publicly legible and politically actionable, thereby conditioning the intensity and expression of skin color stratification across countries (Telles et al., 2025). Finally, the literature also argues that inequality is reproduced not only through treatment and resources but also through interpretive schemas: in contexts where mestizaje narratives historically encouraged class-based readings of disadvantage, individuals may experience unfair treatment yet find it difficult to attribute it unambiguously to skin color versus class, a pattern framed as “dual discrimination” and attributional ambiguity. This ambiguity matters because it can reduce the visibility and contestability of skin-color-based exclusion in everyday life, helping subtle forms of stratification persist even when overt racism is normatively denied (Dixon, 2019; Dixon and Telles, 2017).

Despite these advances in theorizing mechanisms, three gaps remain particularly consequential for theory, measurement, and identification. First, while cross-national studies consistently document that skin color is associated with unequal life chances, the sources of between-country heterogeneity are more often treated as residual “context” than as a theoretically specified moderator. This leaves underdeveloped the proposition that national ethnoracial configurations, understood as historically sedimented and contemporaneously reproduced patterns of intergroup relations that coexist with particular distributions of phenotypes and ethnoracial groups, systematically condition how skin color is converted into socioeconomic advantage and disadvantage (Telles et al., 2015; Telles et al., 2025; Dixon and Telles, 2017). In this article, we therefore operationalize “ethnoracial configuration” as a cluster-based typology that is explicitly designed to approximate both compositional features, such as diversity and the distribution of phenotypes and groups, and historically shaped relational regimes, including the legacies of mestizaje and whitening as frameworks for boundary-making and hierarchy (Telles et al., 2025; Dixon, 2019). Second, although recent work has taken important steps toward more careful empirical strategies in the study of the skin color effect on income, either by pushing further toward causal interpretation or by substantially refining cross-national descriptive architectures, parallel progress remains limited for another key stratification outcome, education, where the evidentiary base continues to rely disproportionately on conventional observational adjustments rather than designs that directly target causal identification (Woo-Mora, 2025; Gómez-Echeverry, 2024; Telles et al., 2015). Third, a further methodological gap concerns measurement: interviewer-rated skin color, including palette-based ratings, is substantively meaningful but also susceptible to interviewer-specific scale use and correlated error, and recent evidence indicates that explicitly modelling interviewer effects can materially improve estimation of skin color gradients, yet this remains uncommon in comparative research practice (Dixon and Telles, 2017; Cernat et al., 2019a).

### 1.1 Skin color and education

Against this backdrop, education is a particularly informative outcome for studying skin color stratification in Latin America because it is a core dimension of well-being and a major gateway through which advantage is converted into occupational status and earnings across the life course (Monk, 2015; Cutler and Lleras-Muney, 2006). The central causal account in the literature is not a single pathway but a sequence: skin color is correlated with historically accumulated class advantages and disadvantages that structure early-life resources and school opportunities, while also shaping how students are perceived and treated within educational institutions, so that unequal investments and unequal treatment can cumulate into durable differences in years of schooling (Telles et al., 2015; Telles et al., 2025). The most influential cross-national evidence in this vein, developed in the Pigmentocracies framework, shows that interviewer-rated skin color measured with the PERLA palette is negatively associated with educational attainment across eight Latin American countries, and that this gradient persists when models incorporate ethnoracial self-identification and standard proxies for social origins, although its magnitude varies markedly by country (Telles et al., 2015; Telles et al., 2025).

Country-focused studies reinforce both the substantive association and the importance of measurement: in Brazil, interviewer-rated skin color remains strongly associated with educational attainment net of parental occupational status and other controls, and it predicts education more consistently than self-classified census race categories (Monk, 2015); in Mexico, a detailed analysis finds a negative education gradient by skin color, with the most robust disparities concentrated at the extremes and attenuating once a rich set of family background and skill-related covariates is introduced (Campos-Vázquez and Medina-Cortina, 2019). Complementary work, including research on Latino populations in the United States, is analytically useful because it clarifies micro-mechanisms that are difficult to observe directly in cross-sectional surveys: within-family comparisons still show lower educational transitions for darker-skinned siblings, and early-school achievement gaps can emerge through teacher-rated behavioral assessments that mediate the relationship between skin color and later test performance, consistent with interpretive and evaluative biases inside classrooms (Ryabov, 2016; Kim and Calzada, 2018). Finally, recent comparative research using harmonized survey data shows that the education gradient by interviewer-rated skin color is not limited to stark contrasts between “light” and “dark” categories. Rather, each step toward a darker skin color is associated with fewer years of schooling and with lower intergenerational educational mobility. This pattern appears both as a within-country gradient, in which darker-skinned individuals attain less schooling than lighter-skinned peers in the same national context, and as cross-national heterogeneity, in which the steepness of that gradient and the level of mobility differ systematically across countries, consistent with the idea that national contexts condition how skin color is translated into educational advantage (Woo-Mora, 2025).

Building on the evidence reviewed above, our contribution to the sociology of stratification is to estimate the causal effect of skin color on educational attainment in Latin America and to assess how this effect varies across national contexts. In continuity with the Pigmentocracies tradition and subsequent comparative research documenting persistent skin color gradients in education, we expect the average effect of skin color on schooling to be negative (Telles et al., 2015; Telles et al., 2025).

H1: Given a set of sociodemographic controls *X*, the Average Treatment Effect (ATE) of skin color (D∈{1,…,11}) on years of schooling (*Y*) is negative and statistically significant.

### 1.2 Interviewer effects

Interviewer-assigned skin color measures, including palette-based instruments, are substantively attractive because they approximate how phenotype is likely to be perceived in face-to-face interactions, yet they are also vulnerable to interviewer-induced error that is both random and systematic. The core concern is that interviewers can differ in how they map observed skin color onto an ordinal scale, generating random rater noise as well as stable “severity” or “leniency” in ratings; in addition, interviewer characteristics (including interviewers’ own skin color) can systematically shift the ratings they assign, and these tendencies can vary across countries and fieldwork settings (Hill, 2002; Hannon and DeFina, 2014; Hannon and DeFina, 2016; Villarreal, 2010; Cernat et al., 2019a; West and Blom, 2017) For studies estimating the effect of skin color on education, this matters because interviewer effects can induce non-classical measurement error in the treatment and can confound interviewer-specific rating practice with local context, especially when interviewers are geographically clustered. This concern is especially relevant in LAPOP, where interviewers record skin color only after completing the interview, so ratings may incorporate not only facial appearance but also cues observed during the interaction. In that scenario, conventional “naïve” models that treat interviewer-assigned skin color as error-free can understate the magnitude of skin color gradients in years of schooling and can also distort cross-national comparisons by mixing substantive heterogeneity with country-specific differences in interviewer practice (Dixon and Telles, 2017; Telles et al., 2015; Sundström and Stockemer, 2022).

Despite the substantive importance of interviewer effects, explicit adjustments for them remain relatively uncommon in comparative survey-based research for reasons that are both data-related and methodological. Many public-use survey files do not include unique interviewer identifiers, or make them difficult to access, which prevents researchers from estimating interviewer-level variation directly. Even when such identifiers are available, key interviewer-level variables are often missing, limiting more refined analyses of how interviewer characteristics shape survey responses. At the same time, interviewer effects are often treated as a secondary methodological issue relative to substantive findings or broader theoretical concerns, and are therefore left unmodeled in applied comparative research (Beullens and Loosveldt, 2016; Sundström and Stockemer, 2022).

These constraints are especially relevant in research on race, ethnicity, and skin color in Latin America. Systematic and comparable measurements of skin color remain relatively scarce, and the few studies that do incorporate interviewer-rated skin color are concentrated in a limited number of national settings rather than in fully comparable cross-national designs. In the specific case of LAPOP/AmericasBarometer, interviewer-rated skin color has been collected for many years and provides the broadest cross-national coverage in the region, but for a long period the unique interviewer identifiers needed to model interviewer-level variation were not consistently released in the public integrated files across waves. As a result, even though the survey collected relevant interviewer information, researchers often could not directly estimate interviewer effects in comparable cross-national analyses. This helps explain why the explicit adjustment for interviewer effects remained uncommon in prior work. Indeed, the analysis by Cernat et al. (2019a, 2019b) relied on the 2010 wave precisely because it provided the information necessary to estimate interviewer-level variation, while earlier work also noted substantial missingness in interviewer-related variables in some country cases, particularly Ecuador and Brazil (Bailey et al., 2014). Taken together, this suggests that the omission of interviewer adjustments is not specific to LAPOP/PERLA-type data alone, but reflects a broader limitation of prior comparative research that is especially consequential in the study of skin-color stratification. These constraints help explain the gap our empirical strategy is designed to address.

The methodological literature therefore points to both design and statistical remedies, including the use of calibrated palettes or electronic devices, explicit modelling that separates interviewer and area components (e.g., cross-classified multilevel specifications), and adjustments that account for systematic interviewer bias (Massey and Martin, 2003; Snijders and Bosker, 2011; Cernat et al., 2019b). In our approach, we mitigate this threat by controlling for interviewer fixed effects, absorbing interviewer-invariant tendencies in rating practice (style, severity, stable perceptual priors). We implement these effects in a way that is consistent with the nested structure of the data (interviewers effectively nested within countries) and we cluster standard errors at the country level to account for within-country dependence and location-specific sources of measurement error. Together, these adjustments should reduce systematic rater-induced bias in the skin color measure and strengthen the credibility of causal inference in the study of educational stratification (West and Blom, 2017).

Consistent with evidence that interviewer-induced measurement error can attenuate estimated skin-color gradients, prior work shows that “naïve” specifications that ignore interviewer effects tend to yield smaller (less negative) estimated associations between skin color and key stratification outcomes, including education and income, than specifications that explicitly adjust for interviewer-related variance (Cernat et al., 2019a; Cernat et al., 2019b). While confidence intervals often overlap (suggesting that differences are not always statistically distinguishable) these results nonetheless imply a directional expectation: once systematic interviewer-specific tendencies in skin-color ratings are accounted for, the estimated effect of skin color should become more pronounced in magnitude relative to an otherwise comparable model that treats interviewer-rated skin color as error-free.

H2: Given a set of sociodemographic controls *X*, the estimated Average Treatment Effect (ATE) of skin color (D∈{1,…,11}) on years of schooling (*Y*) is more negative when interviewer effects are accounted for.

### 1.3 The moderating role of Ethnoracial configuration

Prior comparative research indicates that the stratifying consequences of skin color are not uniform across Latin American countries but instead vary systematically with national “racial regimes” and the institutionalization of ethnoracial boundaries. Using cross-national survey data for the Americas, Bailey et al. (2014) show that the structure of inequality differs sharply across contexts, including whether disparities are better captured by perceived skin color gradients, by categorical race/ethnicity, or by a combination of both. A central implication is that country-level institutions and histories shape how skin color is socially read and politically organized: in settings where Black identities have achieved stronger forms of legal recognition and collective articulation, such as Brazil and Colombia, the link between skin color and stratification may be less pronounced than in contexts where those identities are less institutionalized or politically salient, such as Mexico or Peru (Mitchell-Walthour, 2017; Telles and Paschel, 2014). This institutional dimension also intersects with policy: Brazil’s experience with race-targeted interventions, including affirmative action, is frequently treated as part of the broader configuration in which skin color and ethnoracial categories acquire specific social meanings and distributive implications (Telles and PERLA Team, 2014; Mitchell-Walthour, 2017). From a complementary perspective, scholarship emphasizes that the meaning and social consequences of skin color are context-dependent because classificatory schemas are mediated by census labels and state practices, as well as by national ideologies of mixture and belonging; as a result, the same phenotypic cues may map onto different boundary repertoires and status signals across countries (Loveman et al., 2012; Wade, 2010; Telles and Paschel, 2014). Yet despite broad agreement that these country-level features matter, the literature has rarely developed a unified theoretical framework and corresponding statistical strategy that treats national context as a structured moderator of the skin color effect, rather than as an unspecified residual difference across countries.

Taken together, the evidence reviewed in this section suggests that country-level ethnoracial factors exert a dual influence that is directly relevant for both measurement and stratification processes. On the one hand, national contexts shape how skin color is socially perceived and classified, which matters particularly when skin color is recorded through interviewer assessments, because the mapping from phenotype to scale values is filtered through locally salient racial schemas and classificatory repertoires (Telles and Paschel, 2014; Loveman et al., 2012; Wade, 2010). On the other hand, these same contextual dimensions shape how skin color translates into key stratification outcomes such as education, by structuring the political recognition of darker-skinned identities, the salience of ethnoracial boundaries, and the institutional environments in which schooling opportunities are distributed. Conceptualizing these dimensions jointly as an *ethnoracial configuration*, defined as the distribution of skin color and ethnoracial identities in a country and the historically sedimented patterning of intergroup relations, leads to a clear moderation expectation: in more homogeneous contexts, and especially where ethnoracial diversity is less institutionally recognized through law, measurement, and policy, the causal effect of skin color on educational attainment should be more pronounced, because darker skin is less likely to be counterweighted by recognized categories, collective articulation, or policy interventions that make disadvantage visible and actionable.

*H3:* Given a set of sociodemographic controls *X*, the causal effect of skin color (D∈{1,…,11}) on years of schooling (*Y*) is more negative in countries whose ethnoracial configuration is characterized by greater homogeneity and lower institutional recognition of darker-skinned identities than in more diverse and institutionally recognized configurations.

### 1.4 Social origin and baseline controls

In Latin American stratification research, social origins are routinely treated as the predominant driver of educational inequality, with ethnoracial disparities often interpreted as largely derivative of class reproduction rooted in historically accumulated advantage (Franco et al., 2007; Flores and Telles, 2012). In their analysis, Telles et al. (2015) make this “class versus race” premise empirically explicit by modelling class origins through parental occupational status, measured retrospectively for the household head when respondents were around age 14, thereby capturing inherited stocks of human, cultural, and social capital that structure schooling attainment. At the same time, their results underscore why a robust proxy for social origins is indispensable: parental occupation is consistently positive and highly significant, yet the skin color penalty persists even after accounting for class origins, indicating that educational stratification cannot be reduced to inherited class alone (Telles et al., 2015). For our purposes, parental occupation and maternal education capture social origins through partially overlapping channels, including access to material resources, cultural capital, and home conditions that shape educational opportunities In our setting, maternal education therefore provides a conceptually close and empirically powerful proxy for social origins because it directly indexes educational resources, skills, and home learning environments that shape children’s schooling trajectories (Winkler, 2004). In recent research on this topic, maternal and respondent schooling are strongly correlated, and maternal education is treated as a baseline circumstance for assessing skin color gradients in educational attainment and mobility (Woo-Mora, 2025).

Social origins can shape not only educational attainment but also the way skin tone is socially perceived and recorded by interviewers, which is why maternal education is relevant along both channels. A long-standing Latin American idiom captures this mechanism: “money whitens,” meaning that higher social status and the cues that accompany it (speech, dress, interactional style) may influence how observers classify a person’s skin tone, sometimes leading higher-status individuals to be perceived as lighter and lower-status individuals as darker than their phenotype alone would suggest (Telles and Paschel, 2014; Telles and Flores, 2013; Cernat et al., 2019a). Comparative evidence further indicates that these status–classification dynamics are context-dependent and vary across national racial schemas and institutional environments.

Importantly, this status–classification process should not be interpreted as reversing the primary causal direction examined in this study. We conceptualize skin tone as an early-life exposure to racially stratified social structures, while recognizing that its social perception may be shaped by historically accumulated socioeconomic advantages and disadvantages. The possibility that achieved status influences how skin tone is perceived reflects a socially mediated classification process embedded within broader systems of inequality, rather than a structural feedback loop in which educational attainment determines phenotypic exposure. In this sense, “money whitens” describes how inequality can influence racialized perception, but it does not imply that the causal arrow under investigation runs from education to skin tone.

Two additional covariates, ethnoracial identity (for example: White, Black, Mulatto, Indigenous, etc.) and size of location (for example: rural or metropolitan area), are relevant because both plausibly shape the interviewer’s assessment of skin color and years of schooling. In the Pigmentocracies framework, census-style ethnoracial identification is analytically distinct from interviewer-rated skin color because it embeds national classificatory regimes and boundary logics, and it is also associated with educational attainment net of skin color due to group-based stratification and within-category heterogeneity (Telles et al., 2015). At the same time, ethnoracial identity can affect how respondents are socially read during the interview, thereby covarying with interviewer assessments of skin color in contexts marked by classificatory ambiguity and the well-documented interplay between status, nation, and racial classification (Telles and Paschel, 2014). Size of location is similarly consequential because it captures structural differences in schooling opportunities across rural and urban settings and is routinely included as a predictor of educational attainment in comparative models; it can also correlate with interviewer assessments insofar as interviewers are geographically clustered and local racial schemas and baseline phenotypic distributions differ across settlement types (Telles et al., 2015).

Although age and sex are not typically conceptualized as primary determinants of skin color, they are strong and well-established predictors of educational attainment and therefore belong in the covariate set used to estimate years of schooling. Age captures cohort-linked differences in exposure to expanding (or stagnating) education systems, while sex accounts for gendered patterns in access and progression that shape completed schooling in the region (Franco et al., 2007; Winkler, 2004). Consistent with standard practice in comparative research on skin color stratification, our benchmark study includes age and a female indicator when estimating skin color gradients in years of schooling across countries, treating them as baseline predictors of the outcome that improve comparability and reduce residual variance in educational attainment (Telles et al., 2015).

## 2 Data, variables and methods

### 2.1 The America’s barometer (LAPOP)

We relied on a single set of nationally representative surveys in Latin America: the 2016–2017 AmericasBarometer, collected by the Latin American Public Opinion Project (LAPOP) at Vanderbilt University. The data consist of face-to-face surveys of adults in 18 of the 19 Latin American countries (excluding Cuba). Country samples typically include around 1,500 respondents, with larger samples in the Peru (2,647), Bolivia (1,691), and Chile (1,625). The survey includes measures of respondents’ education, interviewer-rated skin color, self-reported ethno-racial identification, maternal education, and a set of sociodemographic controls. After restricting the data to cases with complete information on the dependent and key independent variables, final analytic samples vary across countries, ranging from 1,033 cases in the Dominican Republic to 2,251 in Peru. We use the 2016–17 wave because it is the most recent AmericasBarometer round that provides comparable information on maternal education for every country in our sample, a key variable for our study.

### 2.2 Variables

#### 2.2.1 Dependent variable

Our dependent variable was respondents’ years of completed schooling, coded from 0 to 18 (top-coded; see Table 1 for descriptive statistics). We use schooling as an indicator of socioeconomic attainment because it allows us to locate nearly all respondents on a common, comparable scale across countries.

**Table 1:** Descriptive statistics.

Variable/category	Valid *N*	*M*	*SD*	%
Continuous variables				
Age	23,163	39.06	15.81	
Skin Color	23,163	4.12	1.54	
Years of Schooling	23,163	10.29	4.25	
Maternal Education	23,163	2.18	2.03	
Interviewers ID	724			
Country				
Mexico	1,183			5.11
Guatemala	1,263			5.45
El Salvador	1,225			5.29
Honduras	1,340			5.79
Nicaragua	1,185			5.12
Costa Rica	1,233			5.32
Panama	1,151			4.97
Colombia	1,292			5.58
Ecuador	1,303			5.63
Bolivia	1,305			5.63
Peru	2,251			9.72
Paraguay	1,115			4.81
Chile	1,298			5.60
Uruguay	1,274			5.50
Brazil	1,076			4.65
Venezuela	1,360			5.87
Argentina	1,276			5.51
Dominican Republic	1,033			4.46
Size of location				
National Capital	5,013			21.64
Large City	4,520			19.51
Medium City	4,281			18.48
Small City	3,360			14.51
Rural Area	5,989			25.86
Sex				
Male	11,589			50.03
Female	11,574			49.97
Ethnoracial identity				
White	6,251			26.99
Mestizo	10,777			46.53
Indigenous	1,980			8.55
Black	1,170			5.05
Mulatto	1,859			8.03
Other	1,126			4.86
Ethnoracial configuration				
White	5,081			21.94
Mulatto	2,109			9.11
Afro-mestizo	3,803			16.42
Mestizo	6,048			26.11
Indo-mestizo	6,122			26.43
Total	23,163			100.00

Descriptive statistics (means/SDs and percentages) were computed only on the complete-case subsample (i.e., observations with non-missing values for all variables of interest). Percentages are calculated over *N* = 23,163 and may differ slightly from 100% due to rounding.

#### 2.2.2 Skin color

Our key treatment variable is interviewer-rated skin color. The use of a skin color measure assessed by interviewers with the aid of an actual color palette allows us to reasonably fix skin tone, arguably the primary physical characteristic associated with race in Latin America (Guimarães, 2012). Interviewer-rated skin color based on a color scale has been widely used in surveys on racial discrimination and racial attitudes in the United States (Gullickson, 2005; Massey and Sánchez, 2010). In Latin America, however, this measurement strategy remains uncommon in large-scale social surveys; notable exceptions include PERLA and LAPOP, which explicitly incorporate interviewer-rated skin color using a standardized palette across countries.

A growing body of scholarship has raised normative and epistemological concerns about the use of skin-color measures in research on racialized inequality. From a normative standpoint, scholars have warned that visual palettes and related measurement techniques may reify historically sedimented racial hierarchies, reproduce colonial and anthropometric logics of bodily classification, and normalize pigmentocratic categories as if they were natural rather than socially produced. These concerns also extend to the research encounter itself, as some critics argue that insufficient attention has been paid to the perspectives of research participants, who may experience such measurements as intrusive, objectifying, or emotionally unsettling. Abel (2026), in particular, argues that claims to “objective” skin-color measurement can obscure the historical links between these tools and earlier traditions of racial science, while also facilitating deterministic or biologized interpretations of the association between skin tone and social outcomes. From an epistemological standpoint, critics further contend that skin tone does not capture the full multidimensionality of race, that reducing racialization to pigmentation may oversimplify complex social processes, and that palette-based or interviewer-rated measures may reflect socially situated perceptions rather than provide direct access to phenotype. Taken together, these critiques underscore the need to clarify what skin-color measures are intended to capture, how they relate to broader processes of racialization, and under what analytical conditions their use can be justified in the study of pigmentocratic inequality (Abel, 2026; Roth et al., 2022; Solís et al., 2025).

In light of these concerns, we make explicit that this study rejects any biological conception of race and does not treat skin color as a proxy for an underlying natural or essential racial reality. Following Roth et al. (2022), we understand race as a socially constructed and multidimensional phenomenon, within which perceived skin color is only one analytically relevant dimension, distinct from self-identification, holistic classification by others, and other racialized physical traits. Our use of the PERLA palette is therefore not intended to reify racial hierarchies or revive the premises of scientific racism, but to document how historically sedimented processes of racialization—shaped by colonial legacies, accumulated disadvantage, and contemporary discrimination—continue to affect educational opportunity in Latin America. In this sense, the object of analysis is not race as biology, nor skin color as an allegedly objective essence, but socially mediated classifications of skin color, since it is precisely such classifications that may activate discriminatory evaluations and unequal treatment. Moreover, our use of the PERLA palette is not intended to be intrusive or threatening. LAPOP’s methodological documentation indicates that the palette was extensively pre-tested in several countries to assess interviewer usability and field coverage, that interviewers apply it at the end of the interview without asking respondents directly, and that the AmericasBarometer operates under routine questionnaire pre-testing, informed-consent procedures, and IRB-approved human-subjects protections. This is also consistent with PRODER survey evidence, where more intrusive optical colorimeters were assessed in focus groups and did not generate substantial respondent discomfort when carefully implemented (Solís et al., 2025). These design features do not eliminate all ethical concerns, but they do indicate that the instrument was implemented with attention to respondent protection and low intrusiveness. More broadly, prior research suggests that the legitimacy of these measures depends on their reflexive and contextually appropriate use. Roth et al. (2022) argue that skin-color instruments are analytically valuable when they help distinguish among different dimensions of race rather than stand in for the concept itself, while Solís et al. (2025) show that acknowledging the limits of existing palettes, refining instruments, and comparing palette-based and optical measures can improve validity without abandoning the substantive study of racialized educational inequality.

A potential concern with interviewer-rated measures is that respondents’ socioeconomic status may bias interviewers’ assessments of skin color. The surveys used here sought to reduce arbitrary variation through a standardized color palette, explicit instructions to assess only facial skin color, and extensive interviewer training. However, LAPOP interviewers record respondents’ skin color only after the interview is complete, without asking respondents directly, which means that assessments may still incorporate cues observed during the interaction. This makes interviewer-related heterogeneity a substantively important measurement issue rather than a merely technical concern.

Because the distribution of skin tones differs substantially across countries, reflecting distinct histories of demographic flows and racial stratification, we standardized the skin color variable within each country using z-scores. This transformation rescales skin color to have a mean of zero and a standard deviation of one within each national sample. Within our causal framework, coefficients on standardized skin color can be interpreted as average partial effects. Specifically, they capture the average causal effect on years of schooling of a one–standard-deviation increase in skin darkness relative to the country mean, holding constant a high-dimensional set of observed confounders flexibly modelled through machine learning. For example, an estimated effect of −1.5 indicates that, on average, individuals whose skin tone is one standard deviation darker than the national mean attain 1.5 fewer years of schooling than they would have attained had their skin tone been at the country average, all else equal.

#### 2.2.3 Ethnoracial configuration

To capture cross-national differences in the broader ethnoracial context in which individual stratification processes unfold, we constructed a country-level measure of *ethnoracial configuration* based on a hierarchical cluster analysis of the 18 Latin American countries included in the sample. This approach allows us to summarize multiple dimensions of ethnoracial structure (phenotypical stratification, demographic composition, and internal heterogeneity) into a parsimonious categorical typology.

The cluster analysis was estimated using a set of country-level indicators derived from the individual survey data. Specifically, we included three families of variables. First, we used the national mean of interviewer-rated skin color, which captures the central tendency of phenotypical stratification in each country. Second, we incorporated a set of variables measuring the national proportion of each ethnoracial group, based on respondents’ self-identification and harmonized into six categories: White, Mestizo, Indigenous, Mulato, Black, and Other. Third, we included a skin color fractionalization index, designed to capture the degree of phenotypical heterogeneity within each country.

A related concern is that the country-level skin-color indicators used in the clustering procedure are based on interviewer-rated assessments and may therefore reflect cross-national variation in classification practices. Unlike the individual-level DDML models, the clustering stage does not allow interviewer effects to be incorporated in the same way, so some interviewer-related heterogeneity may indeed enter the construction of the ethnoracial configurations. At the same time, the implications of this issue differ across the two analytic stages of the paper. In the individual-level models, interviewer-related error can directly bias the estimated relationship between respondents’ skin color and their years of schooling, which is why adjusting for interviewer fixed effects is essential. By contrast, in the cluster analysis, skin-color distributions are used as aggregated contextual indicators rather than as respondent-level treatments in a one-to-one causal relationship. These distributions are not intended to approximate a “true” phenotypic composition, but rather to capture a socially perceived dimension of skin color that is itself relevant to processes of racialization. Under those conditions, interviewer-specific idiosyncrasies are more likely to be attenuated through aggregation, and skin color remains only one component of a broader multivariate characterization of countries. For this reason, the resulting ethnoracial configurations are best understood as heuristic contextual groupings rather than as fixed or definitive classifications. This does not mean that interviewer-related heterogeneity is irrelevant at the country level: it may affect the precise boundaries of the clusters, especially if classification practices vary systematically across national contexts. Even so, the broad configuration emerging from the clustering is consistent with independent historiographic approaches to ethnoracial differentiation in the region, such as Lizcano’s distinction among Indo-European, Afro-Creole, Afro-Mestizo, and Creole country types. This convergence does not eliminate all concerns about measurement error, but it does suggest that interviewer-related heterogeneity is unlikely to fully distort the broader contextual structure captured by the clustering solution.

Following Alesina et al. (2003), the skin color fractionalization index was computed as:

$${\text{FRACT}}_{j}=1-\sum_{i=1}^{N}s_{\mathrm{ij}}^{2}$$

where $s_{\mathrm{ij}}$ denotes the proportion of individuals in skin color category i in country j , and N is the total number of categories. To construct this index, the original 11-point skin color scale was recoded into three substantively meaningful categories—light (1–3), medium (4–5), and dark (6–11)—following the classification proposed by Telles and the PERLA team. The resulting index ranges from 0 (complete phenotypical homogeneity) to 1 (maximum heterogeneity).

For the purposes of the skin color fractionalization index, we rely on a coarse grouping of the PERLA palette, motivated by comparative and analytical parsimony. The aim of this step is not to assume that fixed cut points carry identical social meanings across all national contexts, but to construct a common regional metric of phenotypic heterogeneity that can be used consistently across countries. In this respect, our strategy follows Telles et al. (2015), a foundational comparative reference in this literature, and is also consistent with the broader PERLA project, which repeatedly uses broad light/medium/dark groupings to summarize cross-national patterns of inequality, discrimination, and stratification (Telles and PERLA Team, 2014). This level of aggregation is especially appropriate for our purposes because a fully disaggregated treatment of the 11 tones would reduce the legibility of country-level contrasts by producing sparse or weakly distinguishable distributions across many categories. At the same time, country-specific thresholds would undermine comparability by making the fractionalization index depend on internally relative rather than regionally common criteria. For example, if identical percentiles were used in every country, the category shares would be mechanically the same and the resulting fractionalization index would therefore also be the same across countries. Conversely, if different percentile thresholds were chosen for each country, the index would no longer be based on a common regional scale, undermining meaningful cross-national comparison. Likewise, alternative continuous dispersion measures such as the standard deviation would capture spread rather than categorical composition and would therefore not be directly equivalent to an Alesina-style fractionalization index; they could also be more sensitive to extreme values in countries with small tails at one end of the palette.

More broadly, our emphasis on contextual variation does not require every stage of the analysis to be defined in context-relative terms. On the contrary, because this is an exercise in comparative stratification research, the clustering stage requires variables measured on a common scale across countries so that distinct contexts can emerge as analytically comparable objects. Without that common prior metric, the resulting groupings would not capture historically grounded contextual differences across the region, but would instead reflect country-specific classifications defined on non-equivalent scales. We also note that this recoding is only one component of a broader clustering strategy that additionally incorporates mean skin color and ethnoracial self-identification, so the contextual typology does not rest on this choice alone. At the same time, we recognize that fixed cut points simplify contextual variation in the social meaning of color across countries. For that reason, the resulting index should be interpreted as a coarse comparative indicator of phenotypic heterogeneity rather than as a context-specific map of the social semantics of each tone. This interpretation is also consistent with the logic of PERLA itself: the palette was designed as a common instrument for cross-national comparison and repeatedly used to identify broad comparative gradients rather than country-specific semantic thresholds.

Because the country-level indicators included in the cluster analysis are measured on different scales, all variables were standardized using z-scores prior to clustering. We then estimated a hierarchical agglomerative cluster analysis using Ward’s method, which iteratively merges countries to minimize the increase in within-cluster variance, measured as the total sum of squared deviations. Given that all inputs are continuous, squared Euclidean distance was used as the proximity measure.

Inspection of the dendrogram and application of the largest jump criterion indicated that a five-cluster solution provided the optimal balance between parsimony and internal homogeneity. To assess the substantive validity of this solution, we conducted an auxiliary two-step cluster analysis incorporating Lizcano Fernández’s (2005) qualitative classification of Latin American ethnoracial formations as a categorical input. When constraining the procedure to five clusters, the resulting classifications closely coincided. We therefore retained the hierarchical solution, as it satisfies standard technical criteria while remaining fully consistent with the historical sociology of race in Latin America.

**Figure 1:**
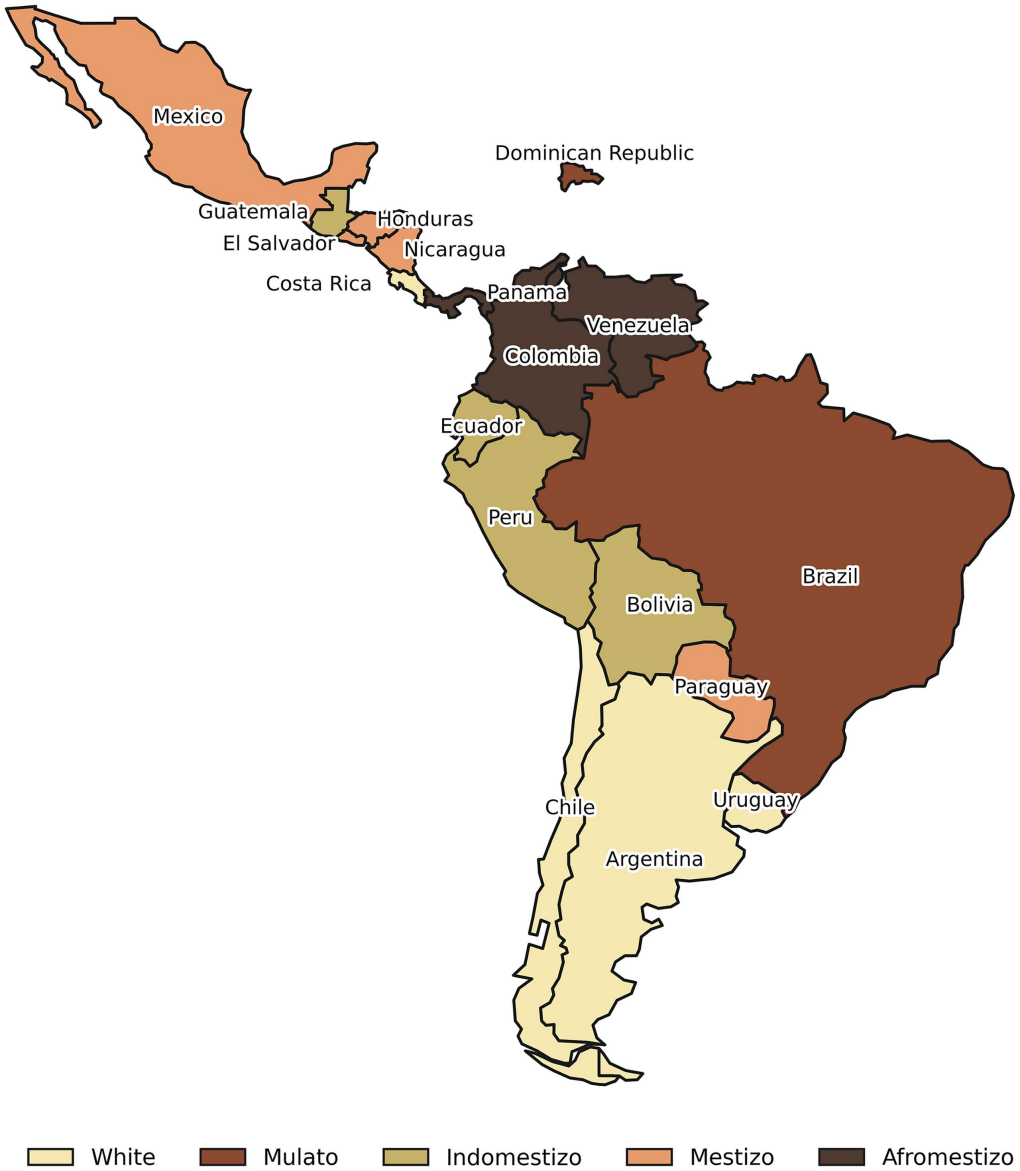
Ethnoracial configuration in Latin America.

The resulting clusters define five distinct ethnoracial configurations (Figure 1), each corresponding to a historically specific pattern of population formation, racial classification, and national identity construction in Latin America, as described by Lizcano Fernández (2005).

The White configuration, comprising Chile, Argentina, Uruguay, and Costa Rica, is characterized by the lightest average skin color, the lowest levels of skin color fractionalization, and the highest proportions of individuals identifying as White. In Lizcano’s terms, these countries approximate *criollo* societies, shaped by early state formation projects that emphasized European ancestry as a central marker of nationhood. This configuration reflects a historical trajectory marked by large-scale European immigration (particularly in the Southern Cone), the demographic marginalization or symbolic erasure of Indigenous and Afro-descendant populations, and the consolidation of national narratives centered on cultural and racial homogeneity. In these contexts, whiteness operates not only as a demographic trait but also as a normative ideal embedded in institutions, citizenship regimes, and conceptions of social order.

The Mulato configuration, composed of Brazil and the Dominican Republic, exhibits the darkest mean skin tones, the highest levels of phenotypical heterogeneity, and the largest proportions of Afro-descendant populations. This configuration closely corresponds to Lizcano’s characterization of *afrocriollo* or *mulato-dominant* societies, where African ancestry is demographically central and historically rooted in plantation economies, slavery, and post-emancipation racial orders. In these cases, racial classification has developed through highly nuanced chromatic distinctions, producing a dense continuum of color categories rather than rigid racial binaries. Lizcano emphasizes that in such societies, mestizaje does not dilute African presence but instead reorganizes it symbolically through gradations of color, making skin tone a particularly salient axis of social stratification.

The Indomestizo configuration, which includes Peru, Bolivia, Ecuador, and Guatemala, is defined by the highest combined proportions of Indigenous and Mestizo populations, alongside relatively dark average skin tones. Historically, these countries overlap with the territories of major pre-Columbian civilizations, such as the Inca and Maya, and correspond to what Lizcano terms *indomestizo societies*. In these contexts, Indigenous populations remain demographically, culturally, and symbolically central, and mestizaje emerges primarily from prolonged interaction between Indigenous communities and colonial or postcolonial elites. Unlike mestizo-dominant societies, indomestizo configurations preserve a visible and politically salient Indigenous presence, often accompanied by enduring forms of ethnic segmentation, territorial inequality, and dual systems of inclusion and exclusion.

The Mestizo configuration, composed of Mexico, Nicaragua, El Salvador, Honduras, and Paraguay, is characterized by a dominant Mestizo population, comparatively lower Indigenous shares than in indomestizo countries, and the second-lightest average skin color. These cases align with Lizcano’s *mestizo societies*, where mestizaje functions as a hegemonic national ideology rather than merely a demographic outcome. Historically, mestizaje in these societies has been promoted as a unifying narrative of national integration, one that symbolically incorporates Indigenous ancestry while simultaneously neutralizing ethnic difference and obscuring internal racial hierarchies. Lizcano underscores that in these configurations, the discourse of racial mixture often coexists with persistent color-based stratification, even as explicit racial boundaries are publicly denied.

Finally, the Afromestizo configuration, formed by Panama, Colombia, and Venezuela, combines relatively dark average skin tones, high proportions of Afro-descendants, and substantial skin color diversity. This configuration reflects Lizcano’s notion of *afromestizo* societies, where European, Indigenous, and African roots are all demographically salient and historically intertwined. These countries are marked by complex colonial histories involving slavery, Indigenous displacement, and sustained racial mixture across multiple regions. As a result, ethnoracial boundaries are neither fully homogenized nor rigidly segmented, producing highly heterogeneous racial landscapes in which color, ancestry, and regional histories intersect in shaping social stratification.

#### 2.2.4 Social origin

Another key independent variable is maternal education, which we use as a proxy for social origin. The AmericasBarometer measures this construct by asking respondents about the highest educational level completed by their mother. We operationalized maternal education as an ordered categorical variable with nine levels: no education; primary incomplete; primary complete; secondary incomplete; secondary complete; technical school incomplete; technical school complete; university incomplete; and university complete. This specification captures meaningful gradients in intergenerational socioeconomic advantage while remaining comparable across countries in the sample.

#### 2.2.5 Interviewer ID and sociodemographic controls

We additionally account for interviewer-specific variation using an interviewer identifier. The survey includes a unique interviewer ID for each interviewer within each country, yielding a total of 724 interviewers across the 18 national samples. This identifier allows us to address potential interviewer-related heterogeneity in survey responses, particularly relevant given the use of interviewer-rated skin color.

The LAPOP survey also collects respondents’ self-reported ethnoracial identification, which we use as an additional key independent variable. The survey classifies respondents into the following categories: White, Mestizo, Mulato, Afro/Black, Indigenous, and Other. To ensure cross-national comparability, we harmonized country-specific response options into these common categories. In particular, when the questionnaire listed multiple Indigenous subgroups in specific countries (such as Amazonian, Aymara, or Quechua), we merged them into a single Indigenous category. Similarly, labels of the same type, such as “Oriental” or “Asian,” were recoded into the Other category. Finally, we collapsed Zambo and Moreno, categories denoting mixed European, Indigenous, and African ancestry, into the Mulato category. Although “Moreno” can sometimes be used in Venezuela to denote Indigenous, Black, or mixed ancestry, we retained this recoding because the term is more strongly associated with Black heritage in official Venezuelan statistical usage (National Institute of Statistics of Venezuela, 2014).

Regarding other sociodemographic controls, age was included as a continuous variable measured in years, ranging from 18 upwards. Sex was operationalized as a binary indicator. Size of location was captured through a set of five dummy variables distinguishing between rural areas (the omitted category), towns or small cities, medium-sized cities, large cities, and metropolitan areas. Descriptive statistics for all variables used in this study are reported in Table 1.

## 3 Methods

Our main objective is to estimate the causal effect of skin color on educational attainment in Latin America and to examine how measurement processes and contextual heterogeneity shape this effect. We proceed in three analytical steps. First, we estimate the average causal effect of skin color on years of schooling, net of a rich set of pre-treatment covariates, thereby establishing the baseline magnitude of pigmentocratic inequality in education. Second, we assess whether this estimated gradient is driven by interviewer-related measurement bias by re-estimating the model with interviewer fixed effects, which identify the effect exclusively from within-interviewer variation in skin-tone assessments. Third, we examine whether the strength of the skin-tone gradient varies systematically across ethnoracial configurations, capturing heterogeneity associated with distinct historical regimes of racial classification and stratification.

Throughout this analysis, we conceptualize skin color not as a manipulable biological trait but as an exposure to racially stratified social structures, following Pearl’s structural causal framework (Pearl, 1995, 2010). In this perspective, skin color operates as an exogenous characteristic that activates socially mediated mechanisms—such as discrimination, segregation, and differential access to institutional resources—which in turn shape educational outcomes. The estimands of interest, therefore, capture the causal impact of these mechanisms of pigmentocracy rather than the consequences of a hypothetical physical intervention on individuals’ phenotypes.

**Figure 2:**
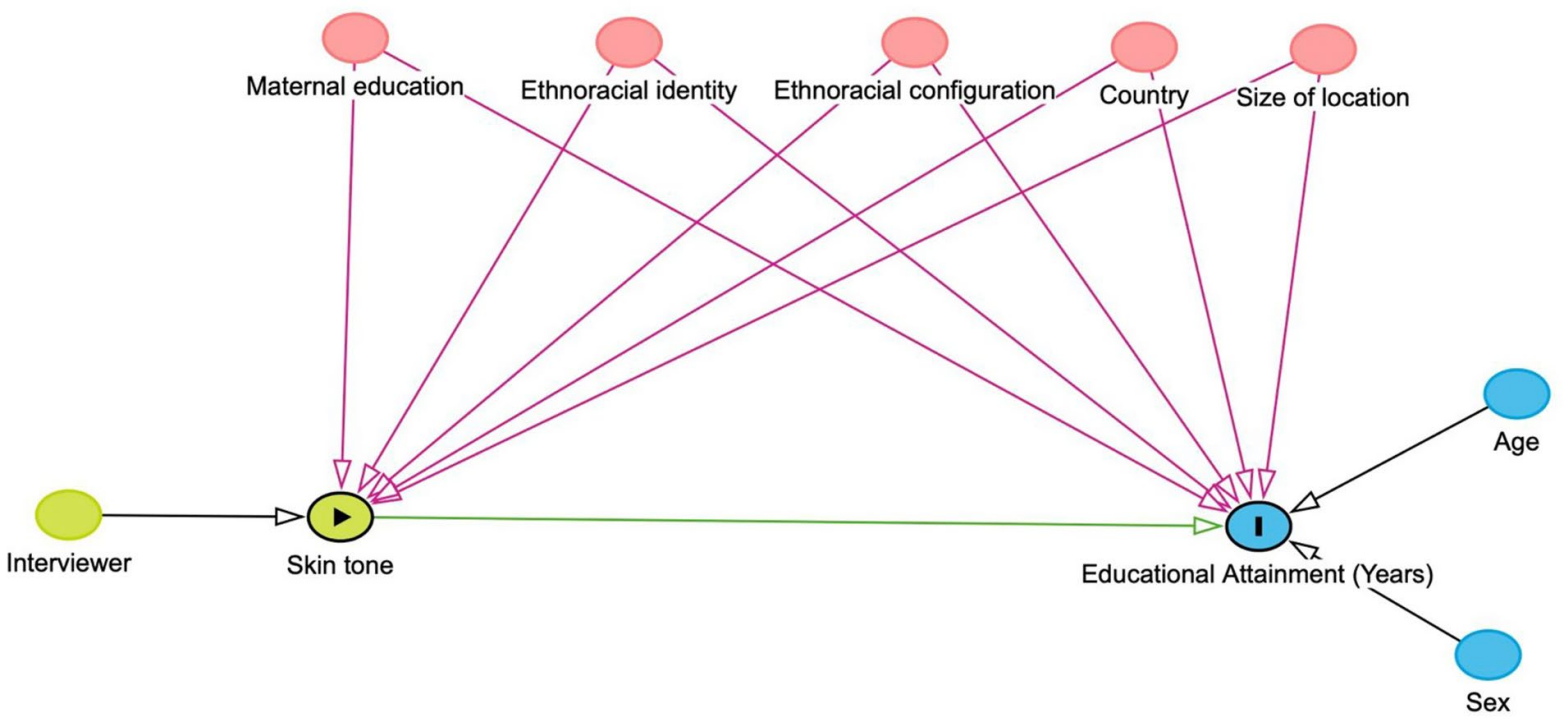
Directed acyclic graph of skin color and educational attainment.

To guide identification, we formalize the assumed data-generating process using a directed acyclic graph (DAG), shown in Figure 2. In this framework, skin color—measured using the PERLA scale and standardized within countries—is conceptualized as an exogenous individual attribute that structures social interactions and opportunities through mechanisms of ethnoracial stratification. Educational attainment is affected by skin color through cumulative processes of differential treatment, expectations, and access to institutional resources, rather than through any biological mechanism.

The DAG identifies several common causes of both skin color and educational attainment that generate backdoor paths between the exposure and the outcome. These include maternal education, ethnoracial identity, ethnoracial configuration, country context, and size of location. Maternal education captures intergenerational transmission of both educational advantage and phenotypic sorting; ethnoracial identity and configuration reflect socially salient regimes of racial classification; country context captures institutional and historical differences across national settings; and size of location proxies urban–rural context, which shapes both educational opportunities and the social distribution of skin color. In addition, interviewer characteristics affect the measurement of skin color but do not directly influence educational attainment, introducing a potential source of measurement-related bias.

Using DAG-based identification criteria, we derive a sufficient adjustment set comprising maternal education, ethnoracial identity, ethnoracial configuration, country, and location size. Conditioning on this set blocks all biasing backdoor paths while preserving the causal pathway from skin color to educational attainment. Interviewer fixed effects are introduced in a subsequent specification to absorb systematic interviewer-specific differences in skin-tone assessment and to evaluate the sensitivity of the estimated effect to measurement processes. Age and sex are included in the empirical models as pre-treatment predictors of educational attainment. Although they are not required for identification—since they do not causally influence skin color in the DAG—they improve the precision of the estimated conditional expectations in the DDML framework and do not introduce post-treatment bias.

A final consideration regarding the DAG in Figure 2 concerns the possibility that social status may influence the perception and classification of skin tone over the life course. As discussed above, this dynamic raises concerns about measurement and potential reverse causality. Within our framework, however, such processes are conceptualized as socially mediated classification mechanisms rather than structural feedback loops in which educational attainment determines phenotypic exposure. The identification strategy outlined here is designed to address these concerns. By adjusting for maternal education, we account for intergenerational socioeconomic position that precedes respondents’ own educational attainment. In addition, specifications including interviewer fixed effects absorb systematic interviewer-level differences in classification practices. Together, these adjustments allow us to distinguish durable pigmentocratic mechanisms from measurement-related bias, thereby preserving the interpretation of skin color as an exposure to racially stratified social structures rather than as an outcome of achieved educational status.

To estimate the causal parameter implied by the identification strategy, we employ Double/Debiased Machine Learning (DDML) in a partially linear specification (Chernozhukov et al., 2018). This approach allows us to flexibly adjust for high-dimensional confounders and complex functional relationships while retaining valid statistical inference for the parameter of interest. DDML is particularly well-suited to our setting, where the adjustment set includes multiple categorical and continuous covariates and where functional form assumptions are difficult to justify *a priori*.

Formally, we estimate the average marginal effect of within-country standardized skin color on educational attainment using a model of the form

Y=θD+g(X)+ε

where Y denotes years of schooling, D denotes skin color (standardized in z-scores within countries), and X represents the adjustment set implied by the DAG. The function g(X) captures the potentially nonlinear and high-dimensional relationship between the covariates and the outcome. We estimate the nuisance functions E[YX] and E[DX] using two alternative learners: (i) a parametric linear regression and (ii) a flexible machine-learning specification implemented via pystacked, using cross-validated lasso over an expanded set of covariates including interactions and higher-order terms. The DDML procedure evaluates all learner combinations and selects the preferred specification based on minimum mean squared error.

This DDML framework provides a unified estimation strategy for all analyses presented in the Results section. We first estimate the baseline average effect of skin color on educational attainment. We then extend the model by including interviewer fixed effects to assess whether the estimated gradient is sensitive to interviewer-related measurement processes. Finally, we estimate heterogeneous effects by allowing the skin-color coefficient to vary across ethnoracial configurations via interaction terms. Across all specifications, inference is based on country-level clustered standard errors, accounting for the cross-national structure of the data and allowing for arbitrary within-country dependence. Under the assumptions encoded in the DAG—most importantly, the absence of unobserved confounding beyond the adjustment set—the DDML estimator identifies the total causal effect of skin color on educational attainment, interpreted as the average change in years of schooling associated with a one–standard deviation increases in skin color. All models are estimated in Stata 19 using the ddml package (Ahrens et al., 2024).

## 4 Results

### 4.1 Causal effects of skin color

Model 1 in Table 2 reports the baseline DDML estimates of the effect of skin color on educational attainment. Across all model specifications, the estimated effect is remarkably stable, indicating that the results are not driven by a particular choice of nuisance-function estimator. To avoid overloading the main text with implementation details, we report in the Appendix Table A1 the stability of the DDML estimates across alternative combinations of outcome and treatment learners. The preferred specification—selected based on minimum mean squared error and using flexible machine-learning learners for both the outcome and treatment equations— shows a strong and statistically significant negative association. The estimand in Model 1 indicates that a one–standard deviation increase in darker skin color is associated with approximately 0.32 fewer years of schooling (*β* = −0.322, SE = 0.026, *p* < 0.001), net of family background, ethnoracial positioning, country fixed effects, and urban–rural context.

**Table 2:** Effect of skin color on educational attainment (years of schooling).

Estimate and estimation details	Model 1	Model 2
Skin Color (country-specific z-score)	−0.32*** (0.03)	−0.45*** (0.03)
Observations	23,163	23,163
Country clusters	Yes	Yes
Cross-fitting (folds)	10	10

Entries report DDML estimates from a partially linear model. Models adjust for maternal education, ethnoracial identity, ethnoracial configuration, country fixed effects, urban–rural context, age, sex, and interviewer (only Model 2). Conditional expectation functions are estimated using a combination of parametric and machine-learning learners with cross-fitting. Standard errors (in parentheses) are clustered at the country level. ***p < 0.001.

In substantive terms, the estimated effect in Model 1 implies a nontrivial educational penalty associated with darker skin color. A difference of one standard deviation on the PERLA scale—a contrast well within the observed range in the data—is associated with approximately 0.32 fewer years of schooling, or 3.8 months of an academic year. Larger contrasts between lighter- and darker-skinned individuals (a 5-point difference in the original PERLA scale) correspond to educational gaps of approximately 1 year of schooling. These magnitudes are substantial in the context of Latin American educational systems, where completion thresholds and credential accumulation are critical for later-life outcomes.

**Figure 3:**
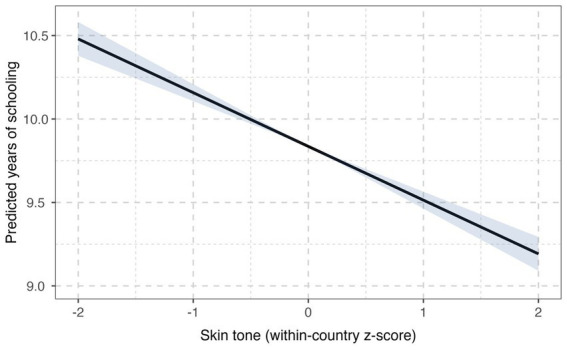
Skin tone gradient in predicted years of schooling. Predicted values are counterfactual expectations implied by the partially linear DDML estimate, evaluated at selected values of standardized skin color, holding the covariate distribution fixed. Shaded areas indicate 95% confidence intervals.

Figure 3 illustrates the estimated relationship between skin color and educational attainment implied by the DDML partially linear model. Rather than plotting fitted values from a conventional linear regression, the figure depicts the counterfactual expectation of educational attainment as skin color varies, holding the distribution of observed covariates fixed. Formally, the curve is constructed from the DDML estimand according to

$$\hat{E}[\mathrm{Ydo}(D=d)]=\bar{Y}+\hat{\theta }\;(d-\bar{D})$$

where Y denotes years of schooling, D is skin color standardized within country, $\hat{\theta }$ is the debiased DDML estimate of the average marginal effect of skin color, and $\overset{ˉ}{Y}$ and $\overset{ˉ}{D}$ are sample means computed over the effective estimation sample. This expression represents the expected level of educational attainment under an intervention that sets skin color to a given value d , abstracting from the influence of confounding covariates.

The resulting curve shows a clear and monotonic gradient, with predicted years of schooling declining steadily as skin color becomes darker. This pattern directly reflects the estimated average causal effect reported in Table 2 and highlights the substantive magnitude of pigmentocratic inequality in educational outcomes across Latin America.

### 4.2 Does the interviewer add measurement bias?

To assess whether the estimated skin-tone gradient is driven by interviewer-related measurement bias, we re-estimate the DDML model including interviewer fixed effects. This specification identifies the effect exclusively from within-interviewer variation in skin-color assessments, thereby absorbing systematic differences in how interviewers translate respondents’ appearance and interactional cues into skin-color ratings. If interviewer bias were responsible for the observed gradient, the estimated effect would be expected to attenuate once interviewer fixed effects are included. Results of this specification are presented in Model 2, in Table 2.

Contrary to this expectation, the estimated effect of skin color becomes larger in magnitude. In the specification with interviewer fixed effects (Model 2), a one-standard-deviation increase in darker skin color is associated with approximately 0.45 fewer years of schooling (*β* = −0.452, SE = 0.029, *p* < 0.001). By comparison, the corresponding estimate in the Model 1 (baseline) without interviewer fixed effects was −0.32 years (*β* = −0.322, SE = 0.026, *p* < 0.001). The increase in magnitude is substantively meaningful, corresponding to an additional loss of roughly 1.5 months of schooling per standard deviation of skin color.

This pattern suggests that interviewer-related heterogeneity does not account for the observed skin-tone gradient in educational attainment. If anything, controlling for interviewer fixed effects reveals a stronger association, indicating that measurement error or interviewer-specific classification practices tend to attenuate rather than generate pigmentocratic inequality in education. Taken together, these results indicate that pigmentocratic stratification in educational attainment is not an artifact of interviewer subjectivity but reflects underlying social processes that persist even when skin-color assessments are compared within the same interviewer.

### 4.3 Heterogeneous effects by ethnoracial configuration

We next examine whether the effect of skin color on educational attainment varies across ethnoracial configurations. To do so, we extend the DDML specification to allow the marginal effect of skin color to differ by ethnoracial configuration, while maintaining the full set of pre-treatment controls and interviewer fixed effects. Formally, we estimate a partially linear model of the form:

$$Y_{i}=\theta_{0}\;{\text{Skin}}_{i}+\sum_{r\neq r_{0}}\theta_{r}\;({\text{Skin}}_{i}\times 1\{{\text{Region}}_{i}=r\})+g(X_{i})+\varepsilon_{i}$$

where $Y_{i}$ denotes years of schooling, ${\text{Skin}}_{i}$ is the within-country standardized PERLA skin-color measure, and $X_{i}$ is the adjustment set implied by the causal DAG, including interviewer fixed effects. The indicator $1\{{\text{Region}}_{i}=r\}$ captures membership in each ethnoracial configuration, with $r_{0}\;$ denoting the reference category (White configuration). In this parameterization, $\theta_{0}$ represents the average marginal effect of skin color on educational attainment in the reference configuration, while $\theta_{0}+\theta_{r}$ gives the corresponding effect for ethnoracial configuration r .

This formulation allows the pigmentocratic gradient to vary flexibly across ethnoracial contexts, while preserving the causal interpretation of the coefficients under the same identifying assumptions as the baseline DDML model. Heterogeneity is thus identified from differential slopes of skin color across configurations, rather than from differences in average outcomes, enabling a direct test of whether historical and structural racial regimes condition the strength of skin-tone stratification in education.

**Figure 4:**
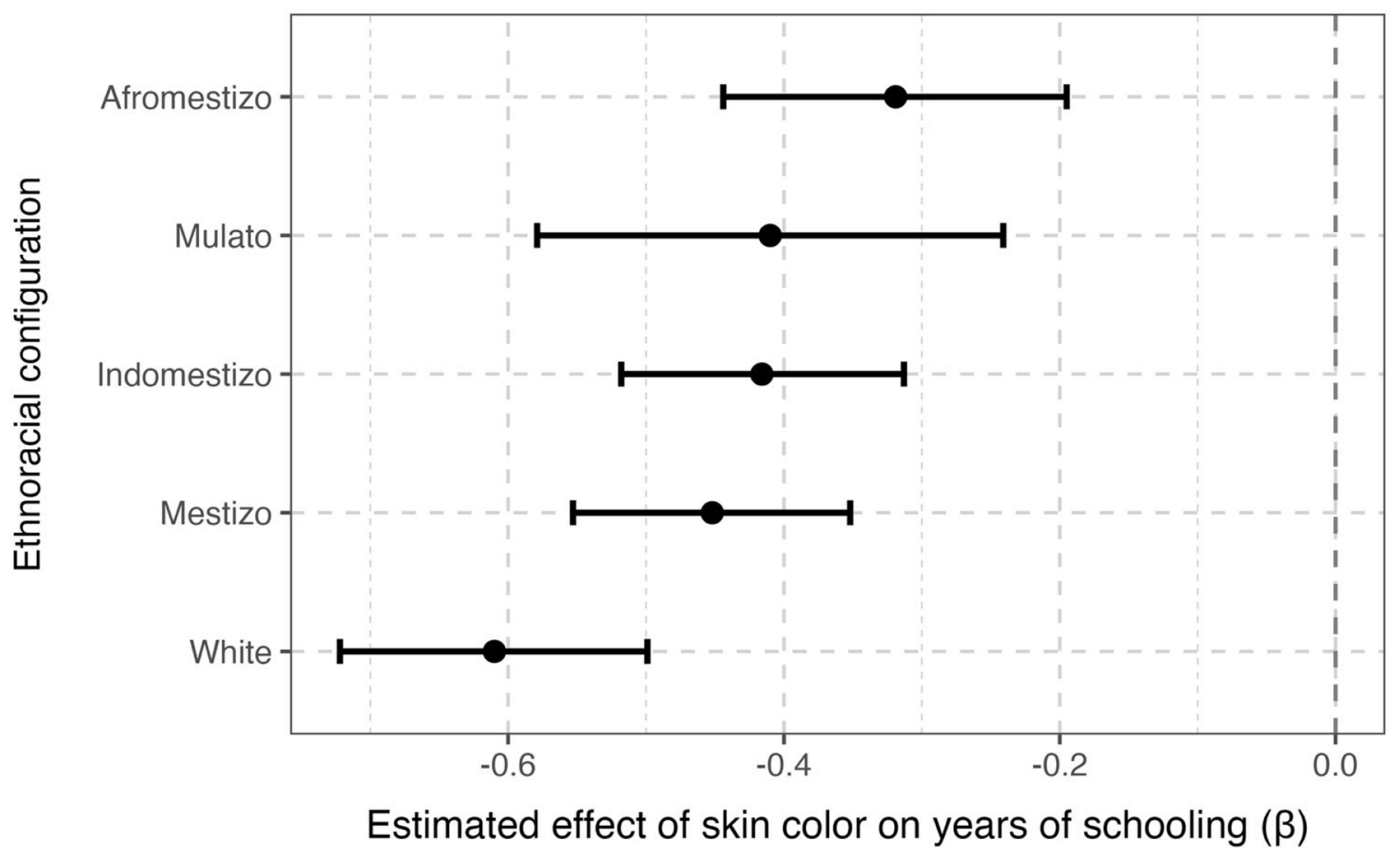
Skin-tone gradient in educational attainment by ethnoracial configuration. Points represent DDML estimates of the marginal effect of standardized skin tone on years of schooling. Vertical bars indicate 95% confidence intervals. Estimates are based on a partially linear DDML model with interviewer fixed effects and country-clustered standard errors.

Table 3 reports heterogeneous DDML estimates of the skin-tone gradient in educational attainment by ethnoracial configuration. In all configurations, darker skin color is associated with significantly lower years of schooling, but the magnitude of the gradient varies meaningfully across contexts. The penalty is strongest in the White configuration (*β* = −0.610, *SE* = 0.057), whereas it is substantially attenuated in the Mulato (*β* = −0.410, *SE* = 0.086), Mestizo (*β* = −0.452, *SE* = 0.051), and Indomestizo configurations (*β* = −0.416, *SE* = 0.052). The gradient is weakest in the Afromestizo configuration (*β* = −0.319, *SE* = 0.063). A joint test of the interaction terms strongly rejects the null hypothesis of homogeneous effects across configurations (*χ*^2^(4) = 14.25, *p* = 0.0065), confirming that the magnitude of the skin-tone gradient differs systematically by ethnoracial regime. Figure 4 visualizes these differences, showing that the skin-tone gradient is steepest in White configurations and weakest in Afromestizo configurations.

**Table 3:** Estimated effect of skin color on educational attainment, by ethnoracial configuration (DDML).

Ethnoracial configuration	Effect of skin color on years of schooling (β)	SE	95% CI
White	−0.610***	0.057	[−0.722, −0.499]
Mulato	−0.410***	0.086	[−0.579, −0.241]
Afromestizo	−0.319***	0.063	[−0.444, −0.195]
Mestizo	−0.452***	0.051	[−0.553, −0.352]
Indomestizo	−0.416***	0.052	[−0.518, −0.313]

Entries report DDML estimates of the marginal effect of skin color (standardized within country) on years of schooling. Estimates are obtained from a partially linear DDML model allowing for slope heterogeneity by ethnoracial configuration. All models adjust for age, age squared, sex, maternal education, ethnoracial identity, size of location, country fixed effects, and interviewer fixed effects. Standard errors are clustered at the country level. A joint test rejects the null hypothesis of homogeneous effects across configurations (*χ*^2^(4) = 14.25, *p* = 0.0065).

To further assess whether the skin-color gradient differs across ethnoracial configurations, we conduct pairwise comparisons of the estimated marginal effects using post-estimation linear combinations. Consistent with the coefficient plot in Figure 3, the penalty associated with darker skin tone is significantly steeper in the White configuration than in the Mulato configuration (difference = −0.81, *SE* = 0.14, *p* < 0.001), indicating a markedly stronger educational gradient at the lighter end of the ethnoracial hierarchy. In contrast, differences among non-White configurations are more modest and generally not statistically distinguishable. The estimated difference between Afromestizo and Mestizo configurations is positive but only marginally significant (difference = 0.13, *SE* = 0.08, *p* = 0.089), while contrasts between Afromestizo and Mulato configurations are small and statistically indistinguishable from zero (*p* = 0.38). Taken together, these results suggest that heterogeneity in pigmentocratic inequality is driven primarily by a sharper gradient within the White configuration, whereas differences among mixed and darker ethnoracial configurations are comparatively limited.

These results indicate that pigmentocratic inequality in education is present across all configurations but is most pronounced in White-dominated contexts and least pronounced in Afromestizo contexts. This pattern suggests that historical and institutional differences in ethnoracial regimes shape not only average educational outcomes but also the strength of skin-color stratification within countries.

## 5 Discussion

In this paper, we deliver three key findings. First, skin color has a causal effect on educational attainment in Latin America under our identification strategy: net of a high-dimensional set of sociodemographic controls (including social origin), darker skin color carries a statistically significant penalty in years of schooling, so that darker-skinned individuals complete fewer years of education than otherwise comparable lighter-skinned peers. Second, this penalty becomes larger in magnitude once we account for interviewer-related heterogeneity in skin-color assessments, indicating that interviewer-induced measurement differences do not generate the pigmentocratic pattern documented in prior work but instead tend to attenuate the estimated causal effect when left unmodelled (Cernat et al., 2019a; Telles et al., 2015). Third, the effect is not constant across national contexts: the causal impact of skin color on schooling is heterogeneous by ethnoracial configuration, varying systematically with cross-national differences in the composition of ethnoracial identities, the distribution and fractionalization of skin-color groups, and the historically sedimented patterning of intergroup relations. Consistent with our expectations, the skin-color penalty is strongest in configurations characterized as White or “criollo” and weakest in more Afromestizo configurations.

Building on the agenda inaugurated by Telles and the PERLA team, the Pigmentocracies project argued that a full account of Latin American stratification requires adding a further layer to class-based explanations, namely the distribution of goods, opportunities, and status along a skin-color gradient that reflects the enduring imprint of colonial racial hierarchies (Telles and PERLA Team, 2014). Drawing directly on that impulse, Telles et al. (2015) showed that interviewer-assessed skin color is often a stronger predictor of educational attainment across Latin American countries than conventional census-style ethnoracial categories (White, Black, Mulatto, etc.), even net of social origins. Yet their seminal contribution also left three questions open that are central to the present study: (1) Does skin color have a causal effect on years of schooling once rich sociodemographic controls are flexibly accounted for, in line with the standards of the credibility revolution? (2) Does the estimated effect persist, and how does it change, when interviewer effects in skin-color measurement are modelled explicitly? (3) Is the effect systematically heterogeneous across countries, such that national ethnoracial configurations, understood as the joint product of compositional diversity and historically sedimented intergroup relations, moderate the skin-color penalty in education?

Recent scholarship has begun to address parts of this agenda, but not the full set of challenges for education. Gómez-Echeverry (2024), for instance, advances the conceptual distinction between ethnoracial self-identification and race-related processes in the study of inequality, yet his empirical focus on income does not resolve the causal and measurement questions surrounding educational attainment. Woo-Mora (2025) provides novel comparative evidence on skin-color gaps in intergenerational educational mobility, but it does not directly target the causal effect of skin color on completed schooling, nor does it develop a theory-driven and statistically explicit account of country-level moderation or incorporate interviewer effects as a systematic source of bias. As a result, and as far as we know, the three questions raised by Telles et al. (2015) have remained only partially answered; our study contributes by addressing them jointly within a single framework that links causal identification, measurement, and cross-national heterogeneity.

Beyond their methodological contribution, these findings also have broader conceptual implications for the sociology of stratification in Latin America, particularly for efforts to understand ethnoracial hierarchization in the region. A first implication is that Latin American stratification can no longer be understood as primarily class-based, with race reduced to a residual legacy of colonialism. For a long time, the region’s stratification literature privileged class as the decisive axis of inequality, while race was often treated as secondary or historically declining. Our findings help place that view in perspective. This is not a simple class-versus-race argument, since both dimensions are deeply intertwined, but neither does that entanglement justify reducing one to the other. Recent comparative work continues to show that racial and ethnic inequality remains a major dimension of socioeconomic inequality in the region, while earlier comparative evidence on pigmentocracies showed that skin color often predicts educational inequality more consistently than census ethnoracial categories (Painter et al., 2019; Telles et al., 2015). In that sense, educational inequality in Latin America is not only class-based but also racialized. At the same time, our more explicitly causal strategy refines those earlier findings by showing that the strength of skin-color stratification cannot be inferred from descriptive cross-national gradients alone, since its magnitude depends on how interviewer heterogeneity and broader contextual configurations are modeled, with important implications for how stratification itself is conceptualized in the region.

A second implication is that the stratifying role of skin color should be understood as relational and contextual, rather than as the expression of a fixed individual attribute. Just as race lacks biological foundation, the social relevance of skin color does not derive from phenotype itself, but from historically specific systems of perception, classification, and valuation that vary across contexts. In this respect, our results resonate with scholarship showing that skin color operates as a socially meaningful status cue and that its significance depends on the position of those making the classification, rather than functioning as a fixed or self-evident trait (Torres et al., 2019). They also invite attention to temporal dynamics within countries: as Muniz and Bailey (2022) show for Brazil, racial classification may shift over time even within a single national setting, which suggests that future research should not only compare countries but also examine how longitudinal processes and changing classificatory regimes reshape the effects of skin color on stratification. Seen in this light, the heterogeneity we identify across ethnoracial configurations points toward different regimes of stratification within Latin America, rather than a single homogeneous pigmentocratic order. Recognizing the existence of a regional pigmentocratic pattern should not flatten the distinct social processes through which color is translated into advantage or disadvantage (Muniz and Bailey, 2022; Telles et al., 2025; Telles et al., 2015).

If these regimes differ in how skin color is socially interpreted and institutionally processed, an additional question concerns how such differences were historically produced. A third implication, then, is that the regimes we identify were shaped not by demographic composition alone, but also by the national discourses through which mixture, whiteness, and diversity were historically narrated and politically organized. Comparative work has shown that ideologies of mestizaje were not uniform across the region: while often presented as national projects of integration, they have also been criticized for encouraging whitening, homogenizing internal diversity, and masking persistent racial hierarchy (Telles and Bailey, 2013). Related work on ethnoracial measurement likewise shows that there is no single regional standard for classifying race and ethnicity; rather, politics and tradition have shaped how countries name, measure, and publicly recognize ethnoracial difference (Telles, 2017). From this perspective, the cross-national variation we observe may reflect not only different demographic histories, but also different discursive formations that shaped how skin color became socially meaningful, how inequality was interpreted, and how particular forms of hierarchy were rendered either natural, muted, or politically contestable across Latin American societies. This interpretation is also consistent with recent comparative evidence showing that ethnoracial hierarchy and group ordering vary significantly across countries in the region (Telles et al., 2025).

A key limitation of our approach is that, although high-dimensional adjustment strengthens internal validity relative to conventional specifications, it cannot eliminate bias from unobserved confounding in a setting where skin color is not randomly assigned. Unmeasured factors that are plausibly correlated with both skin color and schooling, such as cognitive skills, early-life health, or fine-grained indicators of school quality and neighborhood opportunity, could still influence the estimated effect and thus limit a fully causal interpretation (Telles et al., 2015; Woo-Mora, 2025). Relatedly, our contextual controls are necessarily coarse: “size of location” captures major urban–rural gradients, but more detailed geographic information could better approximate unequal access to public goods and services across ethnoracial groups and thereby reduce residual heterogeneity that is currently absorbed in the error term. A related limitation is that the ethnoracial configurations are constructed in part from interviewer-rated skin-color distributions, and although aggregation and the multivariate nature of the clustering likely attenuate interviewer-specific idiosyncrasies, cross-national variation in classification practices may still affect the precise boundaries of the resulting contextual groupings. Finally, our design is oriented toward identifying an average causal effect and its cross-national heterogeneity, not toward fully adjudicating mechanisms: the observed penalty may reflect multiple pathways, including differential parental investments and expectations, segregated school markets, unequal networks and information, or direct discriminatory treatment that accumulates over the life course (Telles and PERLA Team, 2014; Dixon and Telles, 2017; Woo-Mora, 2025). Put differently, some limitations speak primarily to identification (remaining unobservables and limited geographic granularity), whereas others speak to interpretation (the inability to isolate the specific mechanisms through which skin color shapes schooling), and both should be kept in view when assessing what our estimates can and cannot claim.

Despite these limitations, our study advances the literature by bringing the analysis of ethnoracial inequality in Latin America into the broader turn toward causal evaluation in stratification research, while retaining the core insight of the *Pigmentocracies* agenda that skin color constitutes an additional and consequential axis of inequality beyond social origins (Telles and PERLA Team, 2014; Telles et al., 2015). Substantively, our evidence supports a causal interpretation of a skin-color penalty in schooling and, importantly, clarifies why the magnitude of this penalty varies across the region: countries that narrate themselves as comparatively “White” or “criollo,” and that tend to exhibit narrower ranges of publicly recognized ethnoracial diversity, show larger estimated penalties than configurations where diversity is more explicitly acknowledged and where darker-skinned identities have greater organizational and political articulation, particularly in cases such as Colombia (Wade, 2010; Telles and Paschel, 2014; Bailey et al., 2014). In that sense, we move beyond simply noting cross-national heterogeneity by linking it to contextual dimensions emphasized in prior scholarship, including national classificatory regimes and ideologies of mixture and belonging, and the politics of recognition that can facilitate collective claims-making and policy responses, as in Brazil’s experience with race-targeted interventions and affirmative action (Telles and PERLA Team, 2014; Mitchell-Walthour, 2017; Loveman et al., 2012; Wade, 2010). Methodologically, our results also refine the field’s measurement debate by showing that interviewer-related heterogeneity in skin-color assessment tends to attenuate estimated effects when ignored, rather than producing the pigmentocratic pattern mechanically, thereby reinforcing the value of explicit corrections in comparative work (Cernat et al., 2019a, 2019b; Telles et al., 2015).

Looking ahead, this research program would benefit from three extensions that are directly testable. First, improved phenotypic measurement, including broader use of electronic colorimetry where feasible, can strengthen cross-country comparability and help separate phenotype from status-conditioned perception, as recent evidence from Mexico demonstrates (Roth et al., 2022; Telles and Paschel, 2014). Second, mechanism-focused progress will require richer stratification surveys than those currently available in multipurpose instruments such as LAPOP, ideally coupled with experimental designs that can adjudicate specific pathways, for example school-market segmentation or direct discriminatory treatment by skin tone, which have been highlighted in recent contributions (Rivadeneira and Canavire-Bacarreza, 2025; Kamel and Woo-Mora, 2023). Third, to deepen pigmentocracy analyses, future comparative work should move toward a more holistic “traitocratic” approach that considers how skin color operates alongside other embodied and cultural cues, including hair texture and facial features, in shaping social classification and life chances (Hordge-Freeman, 2015; Telles and Flores, 2013; Telles and Paschel, 2014).

In short, our results most directly establish a robust average causal gradient, its sensitivity to interviewer-related measurement heterogeneity, and systematic cross-national variation by ethnoracial configuration, while leaving the precise micro-mechanisms, broader adoption of instrumental phenotypic measurement, and cross-national “traitocratic” extensions as priorities for future research.

## Data Availability

The original contributions presented in the study are included in the article/supplementary material, further inquiries can be directed to the corresponding author/s.
