# Supplementary material for: Beyond pigmentocracy: how country-level ethnoracial configurations shape the effects of skin color on educational inequality in Latin America
Source: Front Sociol. 2026 May 19;11:1786805. doi: 10.3389/fsoc.2026.1786805 (PMC13225987; doi:10.3389/fsoc.2026.1786805)
Supplement: Supplementary file 1 [file Table_1.docx]

# Appendix

| **Table A1.** Robustness of DDML Estimates Across Alternative Learners | | | |
| --- | --- | --- | --- |
| **Outcome learner** | **Treatment learner** | **Skin color coefficient** | **SE** |
| OLS | OLS | −0.324 | 0.026 |
| OLS | ML (stacked) | −0.322 | 0.026 |
| ML (stacked) | OLS | −0.322 | 0.026 |
| ML (stacked) | ML (stacked) | −0.322 | 0.026 |
| **Notes.** Entries report DDML estimates of the effect of skin color on years of schooling under alternative combinations of learners used to estimate the outcome and treatment nuisance functions. Skin color is standardized within a country. All specifications adjust for the same set of pre-treatment covariates, including age, sex, maternal education, ethnoracial identity, ethnoracial configuration, country fixed effects, and location size. Standard errors are clustered at the country level. The preferred specification (minimum mean squared error) uses machine-learning learners for both nuisance components. | | | |
